# The detrimental effects of delay on the endorsement of misleading details for emotionally salient events

**DOI:** 10.3389/fpsyg.2023.1212709

**Published:** 2023-11-22

**Authors:** Datin Shah, Lauren Knott

**Affiliations:** Department of Psychology, City, University of London, London, United Kingdom

**Keywords:** misinformation paradigm, retention interval, memory, emotion, arousal

## Abstract

Previous research has shown that the exposure to misleading information continues its detrimental effect on memory over time for negatively arousing events. However, research has also shown that both high-and low-arousing negative events are vulnerable to distortion from misinformation. Therefore, the present study set out to explore the impact of retention interval on memory for negative (arousing and non-arousing) and neutral events in the misinformation paradigm. Participants were presented with a negative high-arousing, a negative low-arousing, and a neutral scene, and exposed to misleading information for central and peripheral aspects of each scene. Recognition memory for scene details was measured 10 min after misinformation exposure and again after one week. We found that, regardless of the type of detail, the effect of misinformation persisted over time for the negative-arousing event but disappeared one week later for the negative low-arousing and neutral events. The results are explained in relation to adaptive function and theories of source monitoring. The findings of this study provide important forensic implications, especially when we consider the arousing nature of crimes.

## 1 Introduction

False memories occur when one recalls an entirely new experience that never occurred or incorrectly recalls details of an experienced event (Roediger and McDermott, 1995). The distortion of memory has become an increasingly prominent focus of cognitive psychological research, especially with its implications to the applied setting. Understanding the fundamental nature of a reconstructive memory system and the memory errors that can occur from it is of paramount importance to the legal field. We should strive to understand factors that may contaminate eyewitness testimony. To this end, over the past four to five decades, researchers have set out to understand the factors affecting errors in recollection and the mechanisms that cause them (Zhang et al., 2021).

When it comes to research examining the formation of false memories, one of the key laboratory paradigms used is Loftus’ misinformation paradigm (Loftus et al., 1978). In the standard three-stage paradigm, participants are first presented with an event (e.g., in the form of a slide show, video, or a staged event). Thereafter, some participants receive misleading information about the event, typically embedded into a questionnaire or a written narrative. Finally, memory is tested for original event details. The “misinformation effect” occurs when participants falsely report the misleading information in their memory reports as being part of the original event. Since the original research, many factors have been shown to influence misremembering. For example, studies have shown that the size of the misinformation effect increases over longer retention intervals (e.g., Frost, 2000; Frost et al., 2002; Mudd and Govern, 2004). Frost et al. (2002) argued that the reduced number of source cues available after a long delay can make participants more susceptible to misleading information, thereby increasing misinformation errors. In contrast factors such as warning conditions, if given prior to the misinformation presentation, can lead to a decrease in susceptibility (see Loftus, 2005) as we are more vigilant to post-event information discrepancies.

Although the misinformation effect has been used in numerous studies, investigating various key factors, it seems that little research has focused on the influence of emotion on the susceptibility to misinformation. In the legal field, eyewitnesses will be questioned about events that will inevitably be emotionally arousing, particularly serious crimes (e.g., an assault, a theft, or murder) therefore the impact of emotion on misinformation warrants a detailed investigation. Interestingly, the manner in which negative events are encoded and later retrieved elicits conflicting views from the field. For example, research has shown that events containing negative emotional detail are better remembered compared to those containing neutral detail (Hamann, 2001; Talmi et al., 2007). When positive and negative images (taken from the International Affective Picture System [IAPS]; Lang et al., 2008) are shown at study, negative images are better remembered at test (Charles et al., 2003). Similar findings are shown for emotional words, although emotionally arousing taboo words are better recalled when neutral and negative items are matched for relatedness (MacKay et al., 2004; Buchanan et al., 2006). This emotional enhanced effect has been shown immediately after study (Murty et al., 2010; Talmi and McGarry, 2012), and is thought to be primarily due to the attraction of attention during encoding (Sommer et al., 2008; Talmi, 2013), but also over a period of delay. The latter has been attributed to consolidation consistent with the Emotional Synaptic Tagging Hypothesis (Bergado et al., 2011; McReynolds and McIntyre, 2012), with greater activity in the amygdala, hippocampus, and parahippocampus, in addition to visual, prefrontal, and parietal areas (LaBar and Cabeza, 2006; Murty et al., 2010; Dolcos et al., 2012).

However, research has also shown that emotions can impair memory for certain details by producing an emotional memory narrowing effect (e.g., Kaplan et al., 2012). This is a phenomenon whereby one remembers information that is central to an emotional event but has poorer memory for peripheral or background information about the event (Kaplan et al., 2012). According to Easterbrook’s (1959) cue-utilisation theory, an individual has a limited number of cues that they can process at any one time. Therefore, as the arousal of an emotional event increases, attention narrows to the most central/arousing aspects of the event and away from the peripheral/background information (Heuer and Reisberg, 1990; Christianson et al., 1991; Steblay, 1992).

The narrowing effect may be specific to negatively arousing stimuli (e.g., Waring and Kensinger, 2009; Yegiyan and Yonelinas, 2011; Van Damme and Smets, 2014). In a review of evidence on the effects of emotion, Kensinger (2009) showed that a narrowed attentional scope to central/specific details was associated with negative emotion but a broader attentional scope was associated with positive emotion. According to the affect-as-information theory (e.g., Schwarz and Clore, 1983), positive emotion indicates a safe and unproblematic situation that does not require the need for increased attention to specific details, thereby resulting in broader information processing. In contrast, negative emotion suggests a problem that must be dealt with, thus there is a greater need to focus on relevant information within the environment, resulting in narrow item-specific processing.

So it appears that emotionally negative events cause an enhanced memory effect, although potentially leading to impaired memory for certain details. Paradoxically, studies have also shown that negative events are susceptible to distortion from misleading information. Porter et al. (2003) examined whether the effects of misinformation exposure varied with the emotionality of photographic scenes. They found that the endorsement of “major misinformation” (a major peripheral object non-existent in the picture) was most common with negative scenes than with positive and neutral scenes. In addition, Porter et al. (2008) asked participants to try and recall “widely publicised” positively-valenced and negatively-valenced public events. Half of the events were fictitious. It was found that recollection was greater for true-negative than for true-positive events, and greater for false-negative than for false-positive events. Similar findings are true of children recalling emotional false memories too (Otgaar et al., 2008).

Van Damme and Smets (2014) were the first to manipulate the effects of both valence and arousal on suggestibility. The emotional nature of an event can be described by means of (at least) two dimensions: valence and arousal (e.g., Russell, 1980, 2003). They presented participants with high-and low-arousing positive, negative, and neutral photographs. Half of the participants were later exposed to misleading central and peripheral details. They found that, regardless of prior exposure to misinformation, participants were less accurate and endorsed more misleading information for peripheral details associated with the negative events (both high and low in arousal). This indicated that *negative valence* narrowed attention. High arousal improved memory for correct central details, and both negative valence and high arousal inhibited control participants’ tendency to endorse false central detail, however, this effect disappeared with previous exposure to misinformation. Van Damme and Smets suggested that the main parts of the negative scenes may act as attention magnets (i.e., a salient or distinctive part that captures one’s attention; Laney et al., 2003) and that this level of memory narrowing may have been due to the activation of goals associated with the negative emotion. That is, the narrowing effect occurs towards details that are goal-relevant (i.e., the goal-relevance approach; Levine and Edelstein, 2009).

Studies such as those presented above use retrieval tasks with only a short delay. What impact does negative emotion have on the misinformation effect over a longer delay? In veridical memory research, we know that memory for emotional stimuli remains stable or improves over time (e.g., LaBar and Phelps, 1998; Sharot and Phelps, 2004; Wang, 2014; for a meta-analysis, see Park, 2005). In addition, central details seem to benefit most from a lower rate of decay (e.g., Christianson and Loftus, 1987). From an evolutionary perspective, being able to remember an arousing experience over time can help an individual prepare for similar events, and guide future behaviour to approach or avoid such situations (Porter and Peace, 2007; Van Damme and Smets, 2014). To the best of our knowledge, only one study has manipulated testing interval and misinformation exposure to examine their effect on susceptibility to misinformation for emotional events. Porter et al. (2010) presented participants with positive and negative emotional images. Misinformation was introduced to half of the participants with a retrieval task that took place immediately and either 1 week or 1 month later. Regardless of event emotion, they found that overall accuracy for misleading details was lower for misled participants than for nonmisled participants across all retrieval intervals, and misled participants showed a greater reduction in accuracy from 1 week to 1 month compared to control participants. However, negative images (compared to positive) were associated with a greater susceptibility to major misleading details, a pattern found at both immediate and delayed retrieval sessions. Thus, relative to positive emotion, negative emotion heightens suggestibility at least for major misinformation, and this persists over time.

Porter et al. (2008, 2010) argued that negative information is better retained in memory over time but is also vulnerable to distortion from misleading information (*paradoxical negative emotion* hypothesis; Porter et al., 2008). Remembering information from negative events can help individuals to avoid or deal with future dangers (Porter and Peace, 2007). However, negative events are also susceptible to distortion. This has been explained as an adaptive need to retain relevant information concerning negative events from trustworthy sources to ensure one is prepared for future related dangers. Porter et al. (2010) argued that major details indicate a significant change in one’s recollection, thus constituting valuable information that may serve a greater benefit in the future. Consequently, at least for Porter *et al*’s study, major details associated with negative events were more likely to be incorporated into one’s memory reports.

Source monitoring failure may also be used to explain these findings. Source misattributions can most often occur when there are similarities between the original information and the post-event information (Johnson et al., 1993; Mitchell and Johnson, 2000). When participants process the post-event information, they may mentally reconstruct the original event or engage in active rehearsal, thus increasing the overlap between the two sources of information (e.g., in sensory/perceptual characteristics) and strengthening the post-event information (Zaragoza and Mitchell, 1996; Mitchell and Johnson, 2000). Source confusion may be worse for negative high-arousing events relative to neutral and emotionally low-arousing events. Negative high arousing events have been shown to benefit memory consolidation of negative emotional details through the activation of the amygdala and hippocampus (e.g., McGaugh, 2000; Dolcos et al., 2005). Thus, it is plausible to assume that mental visualisations of the post-event information would be more vivid and better integrated into memory for the original event, especially if the availability of source cues fades with time (Frost et al., 2002). We may assume that this would not be the case for negative low-arousing events, although this has yet to be examined.

### 1.1 Present study

The aim of the present study was to examine the impact of delayed retrieval and exposure to misinformation on memory for emotionally negative and neutral events, and central and peripheral aspects of these events. We manipulated arousal in negative emotional images, with a neutral image comparison across a period of delay. Negative events regardless of the level of arousal have been shown to be better remembered than neutral information (e.g., Kensinger and Corkin, 2004), but also be susceptible to misinformation (Van Damme and Smets, 2014). We aimed to explore whether retention interval and misinformation exposure differentially impacted misinformation for high and low-arousing negative events. In addition, memory for central details of negatively arousing events may persist over time more than peripheral details (Christianson and Loftus, 1987; Burke et al., 1992). Central details from negative events and high-arousing events have shown to be vulnerable to prior exposure to misinformation (Van Damme and Smets, 2014), though its effect over time is yet to be seen. Thus, we aimed to systematically study the impact of delayed retrieval on susceptibility to misinformation for central and peripheral aspects of negative events. Based on previous research, we predicted that for the negatively arousing event, the magnitude of the misinformation effect for central details would be similar over time, but would increase for peripheral details. As for the negative low-arousing and neutral events, the misinformation effect for central and peripheral details would increase over time. Finally, we were keen to replicate Porter *et al*’s (2010) findings but with a test for different details at immediate and delayed testing sessions. This would eliminate any concern regarding repeat testing with the same memory test (see Porter et al., 2010). This could affect the interpretation of the memory reports if participants contaminate memory for the event images with test responses from a previous test condition. Considering the above, we believe that the present study is the first to examine the impact of delayed retrieval and exposure to misinformation for central and peripheral details for emotionally negative (both high and low in arousal) and neutral images.

## 2 Methods

### 2.1 Participants

Forty-eight participants (age: *M* = 35.35, *SD* = 14.60, age range = 18–60; sex: 32 females & 16 males) took part in both sessions of the study in return for course credits or a small fee. The study was conducted online. An *a priori* power analysis using MorePower 6.0 (Campbell and Thompson, 2012) indicated that a sample size of between 32 and 80 was adequate to detect medium to large interaction effect (see Paz-Alonso et al., 2013 for similar design and effect size) with a power of 0.80. The participants had English as their first language, normal or corrected-to-normal vision, and were not colour-blind. Participants were recruited via online participant recruitment platforms (Sona and Prolific). City, University of London’s Psychology Research Ethics Committee approved the study and ethical principles were followed.

### 2.2 Design

**Figure 1:**
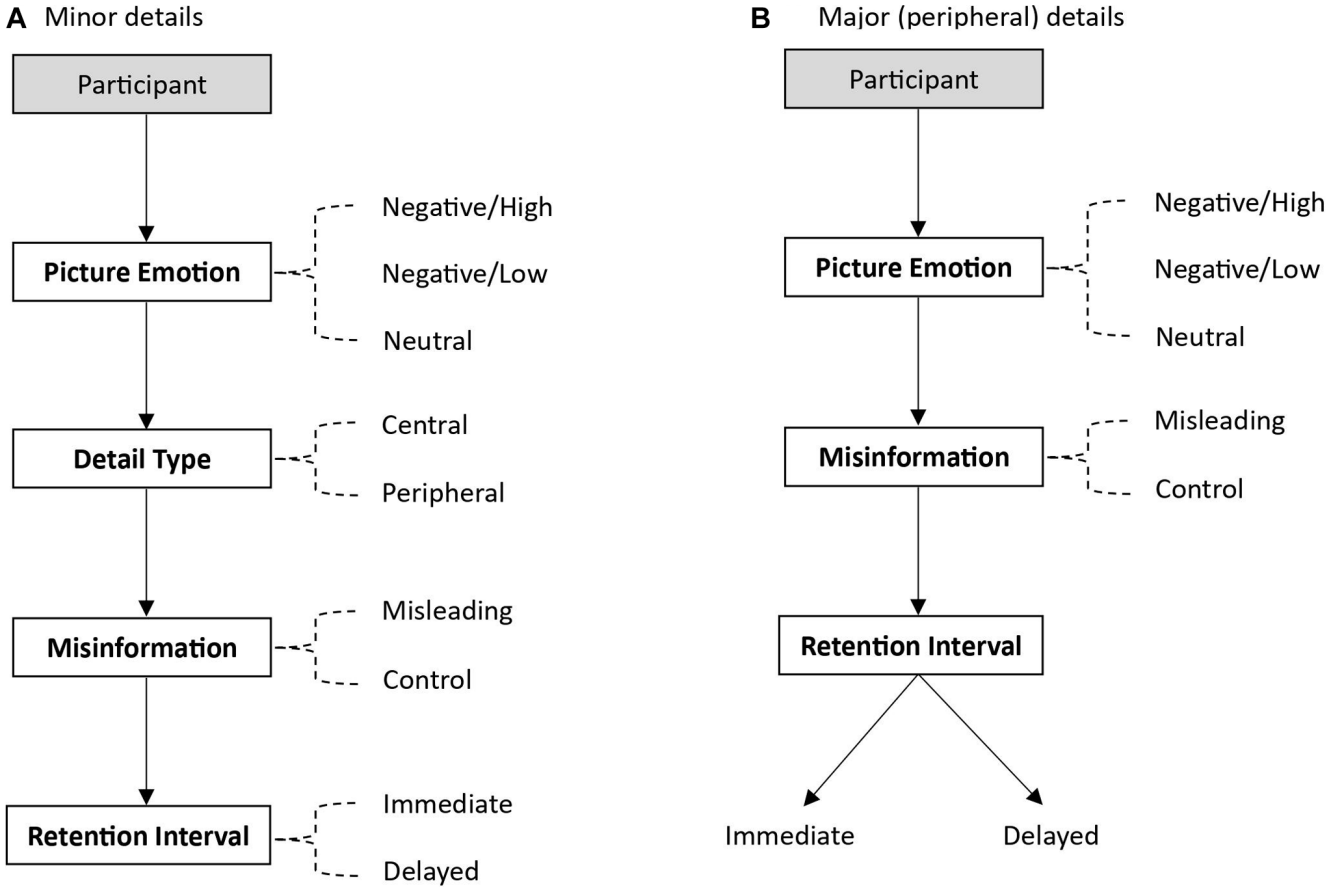
Diagrams representing the experimental design for minor details **(A)** and major details **(B)**. To illustrate the complex designs, the arrows go through the names of the independent variables, with dashed lines indicating all the conditions associated with the variables that participants take part in. For diagram A, the design was fully within-subjects. That is, participants were presented with minor details for all combinations of the levels across the four experimental factors. For diagram B, the design was mixed, with Retention Interval being between subjects. Thus, participants were presented with major peripheral details for all combination of the levels across two experimental factors (Picture Emotion & Misinformation) but were tested on these details either in an immediate or a delayed test.

There were two experimental designs: one for minor details and one for major details. See Figure 1 for a visual representation of the two designs. All participants saw both types of details. For minor details (i.e., details that contradict what was in the picture), the design was a 3 (picture emotion: negative/high vs. negative/low vs. neutral) x 2 (detail type: central vs. peripheral) x 2 (misinformation: misled vs. control) x 2 (retention interval: immediate vs. delayed) repeated measures design. For *picture emotion*, each participant saw three pictures, and the order was counterbalanced: one negative high-arousing (negative/high), one negative low-arousing (negative/low), and one neutral. The presence of *misinformation* was manipulated using five misleading details (two central and three peripheral details) and five control details (i.e., no misinformation was provided for these details; two central and three peripheral details). The misleading and control details were counterbalanced. For *retention interval*, participants completed a recognition test immediately and one week later. As such, the misleading and control details were split between the immediate and delayed recognition tests and counterbalanced. For major details (i.e., a salient peripheral detail that is not present in the picture), there was only one major misleading detail and one major control detail. Thus, the design was a 3 (picture emotion) x 2 (misinformation) x 2 (retention interval) mixed design, with retention interval as a between-subjects variable. Twenty-four participants were in both immediate and delayed conditions. Porter et al. (2003, 2010) only had one major misleading detail in each picture. These details are considered salient and should be noticeable if present, therefore including more than one suggested major detail could make participants aware of the presence of false information and the purpose of the study. The dependent variable was the false recognition of the incorrect answer in the misleading and control questions.

### 2.3 Materials

#### 2.3.1 Picture characteristics

Three pictures taken from the International Affective Picture System (IAPS; Bradley and Lang, 2007; Lang et al., 2008) database were used as to-be-remembered events. The negative high-arousing event was an assault scene (IAPS number: 9254; Valence: 2.03; Arousal: 6.04), the negative low-arousing event was a cemetery scene (IAPS number: 9220; Valence: 2.06; Arousal: 4.00), and the neutral event was a restaurant scene (IAPS number: 2593; Valence: 5.80; Arousal: 3.42).

Central and peripheral details were determined using a pilot task. We used a similar approach to Van Damme and Smets (2014) and Porter et al. (2003), whereby 30 participants (not included in the present study) were asked to draw lines around the central information on each picture. That is, participants were asked to circle on the pictures the area(s) with “the main information that is directly connected to the event, or gist of the event, depicted in the scene” (Christianson, 1992; Luna and Martín-Luengo, 2018). Participants had no time limit for this task. Details were considered central if they fell within the area(s) of the picture, or peripheral if they fell outside of the area(s), by at least 70% of the participants. The central areas judged by the participants included the main characters and objects that were part of the event depicted in the scenes. As such, the central details taken from within these areas included, for example, details about the colour/pattern of clothing worn by the main persons in the scenes, and the type and descriptive features of main objects (e.g., a gravestone). Everything outside of the enclosed lines was considered peripheral information (e.g., the type and number of objects such as streetlamps, the colour of background objects, descriptive aspects of background people) Assessing such details is in line with previous research examining central and peripheral details (e.g., Van Damme and Smets, 2014; Luna and Martin-Luengo, 2018; Jobson et al., 2023).

#### 2.3.2 Post-event questionnaire

The post-event questionnaire (titled “Perception Questionnaire” for the participants) consisted of 10 Yes/No questions about each picture (30 in total). For the questionnaire, we chose ten critical details. Eight of the critical details were *minor* details and two were *major* details. The minor details were selected from the pilot study, whereby four of the details were central (i.e., fell within the central area) and four were peripheral (i.e., fell outside the central area). The major details were *only* peripheral details (thus no central major details were examined). Following Porter et al. (2003), a major detail was defined as a person, animal, or a major object that is falsely suggested to be present in the pictures. Although it is not possible to define the size of the detail since the major details do not exist, in a similar manner to Porter and colleagues, we considered that most, if not all, participants would notice this salient information if present.

For each critical detail, we created a misleading question and a control question. The phrasing of the control questions was kept as similar as possible to the misinformation question except that the misinformation was omitted or the detail was mentioned in a neutral form. An example question with a minor (central) misleading detail concerning the colour of the woman’s top was, “Did you see that the woman’s **brown** top was long-sleeved?” [whereas in fact the top was black; the detail in **bold** was removed in the control version of the question]. Thus, minor misinformation contradicted the details in the pictures. An example question with a major misleading detail concerning the presence of a bird was, “Behind the injured man sitting on the right, did you see the hedge **that had a large pigeon on it**?” [whereas in fact there was no pigeon; the text in **bold** was removed in a control version of the question]. Thus, salient major misinformation added details in the peripheral area. All critical details were never the direct focus of the question; rather, they added extra information in the question.

For each participant, half of the critical details (two central minor, two peripheral minor, and one peripheral major) were misleading, and the remaining half were controls. Thus, the post-event questionnaire contained five misleading questions and five control questions. To counterbalance the combination of detail type and misinformation, two versions of the questionnaire were created. Misleading details in Version A were control details in Version B, and control details in Version A were misleading details in Version B. Therefore, each critical detail served equally often as a misleading and control detail.

#### 2.3.3 Memory test

Recognition memory for the pictures was assessed using 14 two-alternative forced-choice questions per picture. Since the participants were tested both immediately and one week later, two recognition tests were constructed, whereby the 14 questions per picture were split between the two tests. In both Test One and Test Two, two questions probed memory for previously suggested misleading minor details (one central and one peripheral), two questions probed memory for non-suggested control minor details (one central and one peripheral), and two questions probed memory for non-leading details (one central and one peripheral) not previously suggested to all participants. In addition, Test One further included two questions probing memory for the major details (one misleading and one control). Overall, Test One consisted of eight questions per picture and Test Two consisted of six questions per picture. The order of the tests was counterbalanced, such that half of the participants received Test One in the first session and Test Two in the second session. Therefore, for minor details, there was only one question in each test for each combination of detail type and misinformation. For major details, the two questions for major details appeared only in Test One, thus participants were either tested on major critical details in session one or session two (i.e., between-subjects).

For the misleading questions, the two response alternatives were a correct detail (consistent with the picture), and a misleading detail (consistent with the questionnaire). The same response alternatives were used for control questions targeting those details that were misleading for half of the participants. For both the control and non-leading questions, a correct detail and a novel foil were possible answers. An example of a misleading and control question asked during the recognition test is the following: “What colour was the top worn by the woman?” along with two response options: (1) Black [correct] or (2) Brown [misleading/control]. An example of a recognition question targeting a major misleading peripheral detail is the following: “Was there a pigeon in the picture?” along with two response options: (1) No, there was no pigeon [correct] or (2) Yes, there was a large pigeon [misleading/control]. In both tests, participants were instructed to select one of the response alternatives based on their own memory for the pictures. The questions and response alternatives were presented in random order. If they did not know the answer, they were told to make their best guess.

#### 2.3.4 Mood ratings

Pictures may invoke mood changes. Research has shown that mood may impact suggestibility (Forgas et al., 2005; Van Damme and Seynaeve, 2013; Zhang et al., 2021). For example, Forgas et al. (2005) found that positive mood increased misinformation susceptibility whereas negative mood inhibited the endorsement of misinformation. Zhang et al. (2021) found that a positive mood increased misinformation endorsement for neutral scenes. To ensure that there is no confounding effect of a person’s mood on the outcome of the results, we collected participants’ mood ratings at different points during the experiment using Self-Assessment Manikin (SAM; Bradley and Lang, 1994) scales to check for any significant mood changes between sessions. Mood was assessed immediately before picture encoding (session 1) and before each recognition test (sessions 1 and 2).

### 2.4 Procedure

**Figure 2:**
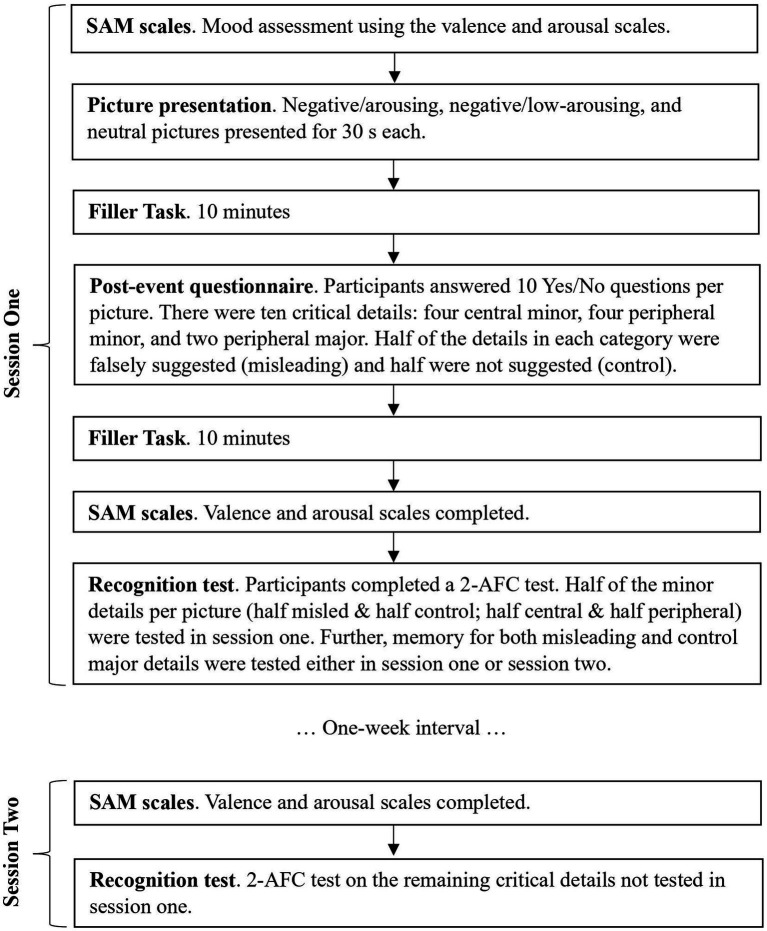
Overview of experimental procedures.

See Figure 2 for a visual overview of the study’s procedure. Participants took part in two sessions. In session one, participants first provided informed consent and then completed the first SAM scale. Thereafter, participants were told that they will be shown some pictures for 30 s each. They were instructed to “Please look at each picture as if you unexpectedly witness the event.” Preceding each picture was a fixation cross for two seconds. The presentation order of the three pictures was counterbalanced. Once all three pictures had been presented, there was a 10-min interval during which time participants completed unrelated filler tasks (i.e., mathematical problems and unrelated anagrams). Thereafter, participants completed the post-event questionnaire in which half of the questions suggested misleading information. The participants were not warned about potential discrepancies between the information in the questions and the picture. The order of the sets of questions about each picture followed the picture presentation order at the encoding stage. After the post-event phase, there was another 10-min interval during which time participants completed reasoning problems. Following this, all participants completed the SAM questionnaire again and the first recognition test. Whether participants received test one or test two in this session depended on the counterbalancing condition that they were randomly assigned to. Before finishing, participants were falsely told that the second session in one week would involve a new set of pictures and they would rate these pictures on two emotional dimensions (valence and arousal). This instruction was used in an attempt to reduce the likelihood of rehearsal in the interim.

Exactly one week later, participants were sent a link for the second part of the study. The link was sent in the morning and participants had until 9 pm on the same day to complete the second part. They first completed the SAM questionnaire to assess their current mood state. Thereafter, they were given the second recognition test. Participants who received test one or two in session one completed test two or test one in the second session, respectively. After completing the recognition test, participants provided demographic information and a debriefing.

## 3 Results

Two participants were removed from all analyses due to failing more than one attention check.^1^ The final sample consisted of 46 participants (age: *M* = 35.48, *SD* = 14.63, age range = 18–60; sex: 30 females & 16 males). For the analysis of major misinformation, there remained 22 participants in the immediate condition and 24 in the delayed condition. The answers in the recognition test were coded dichotomously reflecting false recognition (i.e., correct answer = 0, incorrect answer = 1). The main analyses were conducted on binary false responses to minor and major critical details. An alpha level of 0.05 was used for all statistical tests.

### 3.1 Mood check

To check whether there were any significant changes to participants’ mood between three points in the experiment (Time 1: start of session one; Time 2: immediately before the recognition test of session one; Time 3: start of session two), One-way ANOVAs were conducted on valence and arousal scores separately. Of interest is the difference between Time 1 and Time 3, and between Time 2 and Time 3, since the former represents the start of each session, and the latter represents participants’ mood before each recognition test. No difference in valence scores was found between Time 1 and Time 3 (*p* = 1.00) and between Time 2 and Time 3 (*p* = 0.149) and no significant differences in arousal were found between Time 1 and Time 3 (*p* = 0.203) and between Time 2 and Time 3 (*p* = 0.220).

### 3.2 False recognition

**Figure 3:**
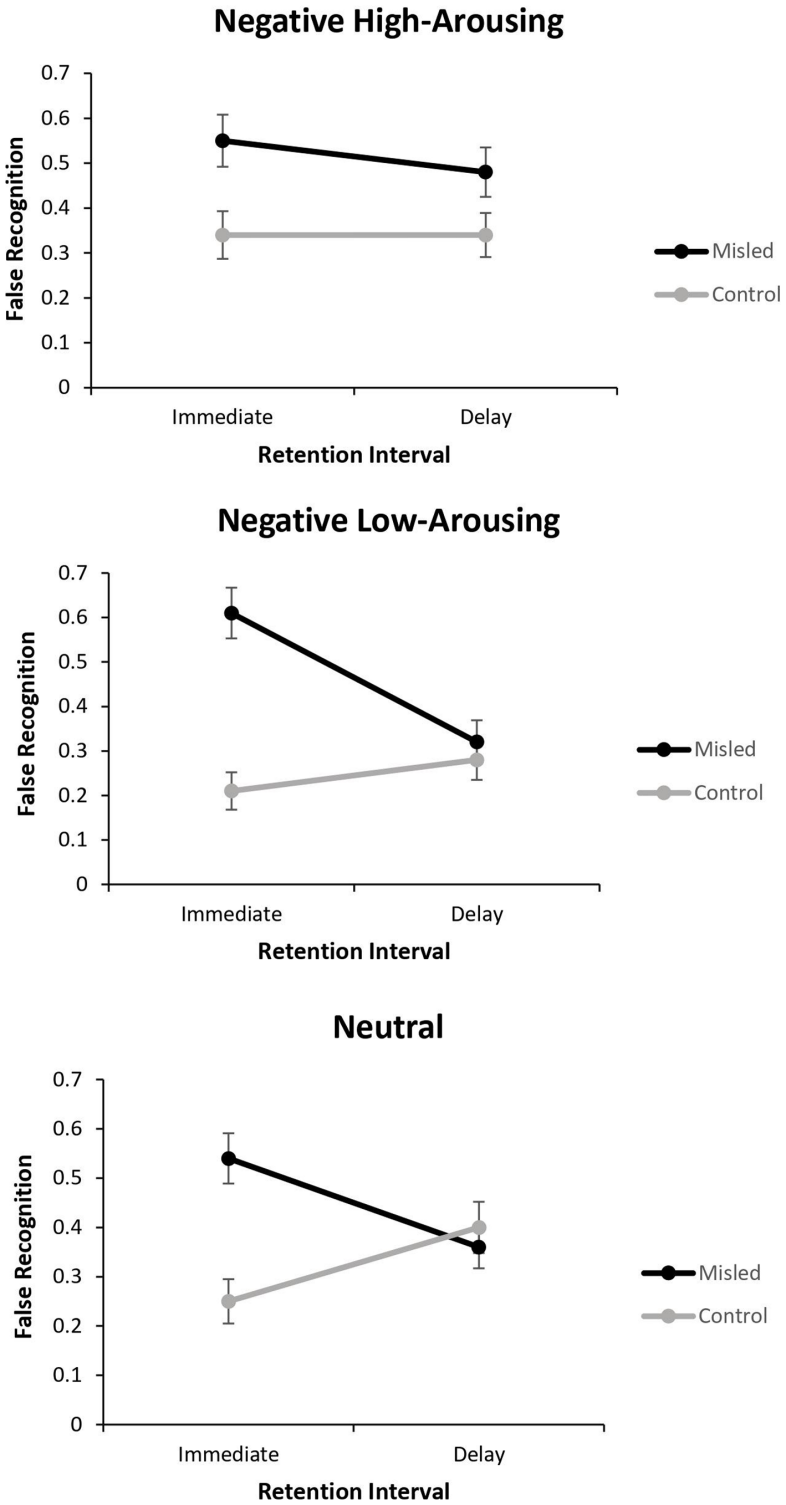
Line graphs showing the proportion of false recognition (out of two binary questions) of the critical minor details for each picture as a function of Retention Interval and Misinformation (Error bars represent the standard error).

The data represented binary responses (0 = correct, and 1 = incorrect). Since log-linear cannot analyse within-subjects data with complex designs, the data were analysed using Generalised Estimating Equations (GEE; Zeger and Liang, 1986). GEE, an extension of the Generalized Linear Model, is an approach that allows for the analysis of repeated measurements and non-normally distributed data. The false recognition responses to misleading and control details were analysed using GEE with a Binomial distribution and log link function.^2^ The repeated factors in the model were picture emotion (negative/high vs. negative/low vs. neutral), detail type (central vs. peripheral), misinformation (misled vs. control), and retention interval (immediate vs. delayed). See Table 1 for means and standard deviations. Post-hoc tests of significant interactions were Bonferroni corrected. Effect sizes for mean differences were estimated using Cohen’s d with the interpretation as follows: small = 0.2, medium = 0.5, and large = 0.8. The means reported in-text are estimated marginal means along with their respective standard deviations. There was a significant main effect of misinformation, Wald χ^2^(1, *N* = 46) = 35.74, *p* < 0.001, *d* = 1.13, and detail type, Wald χ^2^(1, *N* = 46) = 4.50, *p* = 0.034, *d* = 0.39. False recognition was significantly higher for misleading details (*M* = 0.47, *SD* = 0.16) compared to control details (*M* = 0.30, *SD* = 0.14) and for central details (*M* = 0.41, *SD* = 0.15) compared to peripheral details (*M* = 0.35, *SD* = 0.16). There was also a significant retention interval x misinformation interaction, Wald χ^2^(1, *N* = 46) = 9.74, *p* = 0.002, and a picture emotion x retention interval x misinformation interaction that approached significance (see Figure 3), Wald χ^2^(1, *N* = 46) = 5.92, *p* = 0.052. There were no further main effects (Wald χ^2^’s < 5.02, *p*s > 0.081), two-way interactions (Wald χ^2^’s < 2.86, *p*s > 0.239), three-way interactions (Wald χ^2^’s < 2.78, *p*s > 0.133), and a four-way interaction (Wald χ^2^ = 1.24, *p* = 0.537). Because the three-way interaction approached significance and was of interest to our aim to understand the impact of misinformation on memory for negative emotional events over time, we explored this further.

**Table 1:** Descriptive statistics for the false recognition of misleading and control details as a function of picture emotion, detail type, misinformation, and retention interval.

Retention interval	Immediate testing				Delayed testing			
Misinformation	Misleading		Control		Misleading		Control	
	M	SD	M	SD	M	SD	M	SD
Central details								
Negative/High	0.59	0.50	0.33	0.47	0.46	0.50	0.41	0.50
Negative/Low	0.67	0.47	0.22	0.42	0.35	0.48	0.39	0.49
Neutral	0.59	0.50	0.22	0.42	0.43	0.50	0.39	0.49
Peripheral details								
Negative/High	0.52	0.51	0.35	0.48	0.50	0.51	0.26	0.44
Negative/Low	0.54	0.50	0.20	0.40	0.28	0.46	0.17	0.38
Neutral	0.50	0.51	0.28	0.46	0.28	0.46	0.41	0.50

M and SD refer to Mean and Standard Deviation, respectively.

For the negative/high picture, there was a significant main effect of misinformation, Wald χ^2^ (1, *N* = 46) = 9.51, *p* = 0.002, *d* = 0.67, but no significant effect of retention interval (*p* = 0.458) nor interaction (*p* = 0.507), suggesting that the size of the misinformation effect was similar at both immediate and delayed sessions, and no differences in the false recognition of misleading and control details over time. For the negative/low picture, there was a significant main effect of misinformation, Wald χ^2^ (1, *N* = 46) = 20.18, *p* < 0.001, *d* = 0.93, but not retention interval, Wald χ^2^ (1, *N* = 46) = 2.86, *p* = 0.091, *d* = 0.35. However, there was a significant interaction, Wald χ^2^ (1, *N* = 46) = 9.54, *p* = 0.002. A misinformation effect was found at immediate testing (misleading: *M* = 0.61, *SD* = 0.39, control: *M* = 0.21, *SD* = 0.28), Wald χ^2^(1, *N* = 46) = 36.76, *p* < 0.001, *d* = 1.18, but not at delayed testing (misleading: *M* = 0.32, *SD* = 0.33, control: *M* = 0.28, *SD* = 0.31), Wald χ^2^(1, *N* = 46) = 0.19, *p* = 0.661, *d* = 0.12. There appears to be a decrease in false recognition of the misleading details over time. For the neutral picture, there was a significant main effect of misinformation, Wald χ^2^(1, *N* = 46) = 6.70, *p* = 0.010, *d* = 0.58, but not retention interval, Wald χ^2^(1, *N* = 46) = 0.01, *p* = 0.905, *d* = 0.04. However, there was a significant interaction, Wald χ^2^(1, *N* = 46) = 10.97, *p* < 0.001. Similar to the negative/low picture, a misinformation effect was found at immediate testing (misleading: *M* = 0.54, *SD* = 0.35, control: *M* = 0.25, *SD* = 0.31), Wald χ^2^(1, *N* = 46) = 17.72, *p* < 0.001, *d* = 0.88, but not at delayed testing (misleading: *M* = 0.36, *SD* = 0.29, control: *M* = 0.40, *SD* = 0.35), Wald χ^2^(1, *N* = 46) = 0.43, *p* = 0.514, *d* = 0.12. It appears that misinformation continued to influence memory performance over time for the high-arousing negative event, but for the low-arousing events, there was no significant negative impact of misinformation on memory over time; in fact, false recognition of the misleading details decreased over time. Although detail type did not interact with this effect, Table 1 suggests that this was more apparent in the peripheral compared to central detail type.

Based on Porter et al. (2003, 2010) and Van Damme and Smets (2014), differences in the endorsement of the major misleading details across negative and neutral pictures over time were investigated. To do so, the factors picture emotion, misinformation, and retention interval, with between-subjects on the last factor, were submitted to a Generalised Estimating Equation analysis. See Table 2 for means and standard deviations. There was a significant misinformation effect, Wald χ^2^(1, *N* = 46) = 16.96, *p* < 0.001, *d* = 0.80. Furthermore, there was also a significant misinformation x retention interval interaction, Wald χ^2^(1, *N* = 46) = 10.31, *p* = 0.001. At immediate testing, false endorsement rates were higher for misleading major details (*M* = 0.53, *SD* = 0.35) compared to control major details (*M* = 0.13, *SD* = 0.21), Wald χ^2^(1, *N* = 22) = 29.07, *p* < 0.001, *d* = 1.39. However, this misinformation effect was no longer significant at delayed testing (misleading: *M* = 0.33, *SD* = 0.32, control: *M* = 0.28, *SD* = 0.25), Wald χ^2^(1, *N* = 24) = 0.45, *p* = 0.501, *d* = 0.17. There were no further significant main effects (Wald χ^2^’s < 0.69, *p*s > 0.710), two-way interactions (Wald χ^2^’s < 2.17, *p*s > 0.339), and a three-way interaction (Wald χ^2^ = 0.35, *p* = 0.839).

**Table 2:** Descriptive statistics for the false recognition of major details as a function of picture emotion, misinformation, and retention interval.

Retention interval	Immediate testing				Delayed testing			
Misinformation	Misleading		Control		Misleading		Control	
	M	SD	M	SD	M	SD	M	SD
Negative/High	0.59	0.50	0.09	0.29	0.33	0.48	0.25	0.44
Negative/Low	0.45	0.51	0.18	0.40	0.25	0.44	0.29	0.46
Neutral	0.55	0.51	0.14	0.35	0.42	0.50	0.29	0.46

M and SD refer to Mean and Standard Deviation, respectively.

## 4 Discussion

Although extensive research has examined factors that increase and decrease the extent to which we are susceptible to misleading information regarding event recall, there are still questions to be answered regarding the impact of affective factors and how they influence memory distortion. The present study aimed to explore the impact of delayed retrieval and susceptibility to deception for negative/high arousal, negative/low arousal and neutral events, and based on previous emotion memory literature, whether there would be differential effects on memory distortion for central and peripheral details (Kaplan et al., 2012). Although previous research has examined the impact of delay on valanced stimuli, arousal was high for negative and positive images (Porter et al., 2010). To understand the role of arousal on memory distortion over time, participants were presented with a negative high-arousing, negative low-arousing, and neutral scene, followed by exposure to misleading central and peripheral details. Recognition memory was measured shortly after misinformation exposure and one week later.

For the negative high-arousing event, we found, regardless of detail type, a misinformation effect that persisted over time. The magnitude of this effect was medium. Such a finding fits with the paradoxical negative emotion (Porter et al., 2008) hypothesis. This predicts that negative information will be remembered well over time, but can be associated with a greater susceptibility to distorting misleading information relative to other emotional events. This is because retaining memory of negative arousing events can be of adaptive significance (Porter et al., 2008) but it is also adaptive to incorporate all relevant information about negative events from trustworthy sources to further prepare for and/or avoid similar “dangerous” events in the future (Porter et al., 2008). Consistent with this, we found continued susceptibility to misinformation for the negative arousing events over time.

Such outcomes may also be explained based on source confusion. Post-event misinformation associated with the negative arousing event may have a strong memory trace and be more integrated into the original event, making source monitoring difficult. When answering the post-event questions, participants likely engage in the reconstruction of the original event and the rehearsal and visualisation of the misleading information (Johnson et al., 1993). This increases the overlap between memory for the original event and memory for the post-event information, consequently increasing source confusion. This has been empirically demonstrated in previous research (e.g., Dobson and Markham, 1993; Zaragoza and Lane, 1994). Misleading information about the negative low-arousing and neutral events can also be accompanied by mental visualisations. However, since arousal has been shown to benefit memory consolidation of negative information through the activations of the amygdala and hippocampus (e.g., McGaugh, 2002; Dolcos et al., 2005), it is plausible to assume that mental visualisations of the post-event information would be vivid, better integrated into memory for the original event, and better remembered over time. Consequently, misinformation may continue to affect memory for a negative arousing event due to source confusion, especially if the availability of source cues fades with time (Frost et al., 2002).

For the negative low-arousing and neutral events, the effect of misinformation at immediate testing (with a large effect size) disappeared after a delay. This was driven by a significant reduction in the recognition of misleading details after one week. Using an activation-based explanation (e.g., Source of Activation Confusion Model; Ayers and Reder, 1998), we could argue that such an effect occurs because the concept’s strength (e.g., original or misleading detail) decays over time. Therefore, a stronger activation of the recently presented misinformation relative to a weaker activation of the original detail may lead to source misattribution errors of the activated concept. When testing after a short interval, misinformation receives more activation than the original detail because of its recent exposure, thus the original detail is less likely to be retrieved. At delayed testing, however, memory traces for both details are weaker, but the strength of the misinformation item is roughly equivalent to or below that of the original item’s strength (Ayers and Reder, 1998; Lustig et al., 2004). The misleading information has a less distortive effect on memory at one week because its recency advantage is reduced and is thus less accessible to memory. Therefore, the original detail receives more activation and is subsequently retrieved (Ayers and Reder, 1998).

Overall, the reduction in the endorsement of misleading information associated with the negative low-arousing and neutral scenes after one week may be due to the reduced accessibility of the misleading information and greater activation of the original information over time. One could ask why this would not be the case for the negative high-arousing event. However, there are at least two possible reasons for why the spontaneous recovery of the original information did not occur for the high-arousing event. First, as mentioned earlier, the processing of misleading information and its integration within the original event may be stronger through the reconstruction of the original event. Thus, it is plausible to assume that there would be greater source confusions associated with the negative high-arousing event, particularly as we have suggested that cues fade over time (Frost et al., 2002). Second, high arousing information specifically benefits from long-term consolidation (e.g., Kensinger and Corkin, 2004). It may be that the visualisation of the post-event information with the negative-arousing event increases emotional arousal, thereby enhancing the encoding and consolidation of the misleading information and memory over time. Together, misleading information continues to interfere with memory for the negatively arousing event, thereby preventing an increase in correct recognition after a period of delay.

According to our analysis, the effect of retention interval and misinformation on memory for negative and neutral events did not significantly vary for central and peripheral details. Although note the contribution of peripheral details to the reported three-way interaction and therefore the need for future research to continue examining central and peripheral memory. Research has shown that negative events in general cause memory narrowing and that the presence of misinformation increases susceptibility to central misinformation (Van Damme and Smets, 2014). In addition, central information in an arousing event may specifically benefit from long-term consolidation (Christianson, 1992; Park, 2005). Based on these previous findings, we rationalised that retention interval could affect memory for central and peripheral misleading details for different emotional events. Although our findings did not support this rationale, previous misinformation studies have reported mixed results regarding the effect of emotion on memory for central and peripheral misinformation (see Sharma et al., 2022, for a review). This could be attributed to methodological variations between the studies (e.g., the type of memory test, and the way central and peripheral details are determined). Our findings support Porter et al. (2003), who found no significant difference across emotional and neutral scenes. However, future research can determine whether our finding, irrespective of detail type, is a genuine result or an artefact of the study’s design/procedure.

Turning briefly to major misinformation. Porter et al. (2010) found that major (peripheral) details associated with moderate-to-high arousing negative events were vulnerable to misinformation, which persisted over time. Although we saw a misinformation effect for major misinformation details at immediate testing with a large effect, this disappeared after a period of delay, and this did not differentiate across emotional picture conditions. We were unable to replicate negative emotion’s specific susceptibility to “major misinformation” details. Two limitations should be mentioned. First, as this was treated as a between-participants factor due to methodological constraints, our sample size was low for analysing major misinformation. Second, there are procedural differences between these studies, including the type of test, definitions for central/peripheral details, and images used. The misinformation literature is fraught with procedural differences and understanding the impact of those differences in relation to the impact of emotion on memory distortion is work for future research.

To conclude, we found that misleading information continued to distort memory for a negatively arousing event over time, whereas memory performance improved for the negative low-arousing and neutral events. This has important applied implications for the development of false memories in forensic/legal settings. Eyewitnesses typically experience events that are negatively valenced and highly arousing (e.g., a robbery or an assault). They may also be exposed to misleading information about the events from, for example, other witnesses or the media. Indeed the latter point has some significance regarding the impact of conformity to misinformation from certain sources, such as those with perceived high credibility, intelligence, and authority on misinformation endorsement (e.g., Thorley, 2015; Mojtahedi et al., 2020). Furthermore, eyewitnesses may be asked to recall the event immediately after experiencing it or a few hours to weeks after the event (Neubauer and Fradella, 2011). Our findings highlight the continued detrimental impact of misinformation on memory for a negatively arousing event over time. Interestingly though, if the event is low arousing any impact of misleading information may not have a prolonged effect. Finally, we accept that there are some limitations to the present research. First, witnessing a photograph of a traumatizing scene is different from witnessing a real-life crime. Second, the use of forced-choice recognition tests may not reflect most recollections of real-life events where eyewitnesses are less likely to be forced to respond using a set number of responses (though see Howe et al., 2010). Future research can aim to use open questions to reduce the possible impact of correct guessing (Loftus et al., 1985) and response biases (Zaragoza et al., 1987) through the elimination of written cues, thereby increasing the probability of detecting the misinformation effects. Third, in line with previous misinformation research (e.g., Porter et al., 2003, 2010; Peace and Constantin, 2016), the pictures were chosen based on the normed valence and arousal from the IAPS database. Since these ratings were not collected in the present study, the manipulation of valence and arousal was not directly confirmed. Nonetheless, the current study provides insights into the potential impact of arousing negative events and the influences on susceptibility to misinformation for such events. This appears to be specific to arousing negative details and not negative valenced events in general. Given that our research suggests that people who view a highly disturbing scene are far more prone to incorporate misinformation into their memory relative to other scenes, suggests that despite the level of complexity of the event, it is essential that improper questioning techniques be avoided in practise to reduce the problem of inaccurate testimony.

## Data availability statement

The raw data supporting the conclusions of this article will be made available by the authors, without undue reservation.

## Ethics statement

The studies involving humans were approved by Psychology Ethics Committee/City, University of London. The studies were conducted in accordance with the local legislation and institutional requirements. The participants provided their written informed consent to participate in this study.

## Author contributions

The research design, data collection, analysis, and the initial full draft of the manuscript was conducted by DS. All authors contributed to the article and approved the submitted version.

## Footnotes

^1^Typically used for studies conducted online to ensure engagement. There were two attention checks during the picture presentation stage but between stimulus, and one during the post-event stage.

^2^For the purposes of comparability and consistency with relevant previous research (e.g., Porter et al., 2010; Van Damme and Smets, 2014; Jobson et al., 2023), we also performed an ANOVA on binary data. Previous research has conducted an ANOVA on such binary data (e.g., Porter et al., 2010 and Peace and Constantin, 2016, on major misinformation data; Sutherland and Hayne, 2001,). By also using a similar statistical approach, this can help to determine whether the effects found in previous research are also found in the present study. The findings from the ANOVA analysis were similar to those obtained using GEE, and are provided in full as a supplementary material for those interested. Both analyses lead to similar outcomes.
