# Supplementary material for: The detrimental effects of delay on the endorsement of misleading details for emotionally salient events
Source: Front Psychol. 2023 Nov 22;14:1212709. doi: 10.3389/fpsyg.2023.1212709 (PMC10703489; doi:10.3389/fpsyg.2023.1212709)
Supplement: Supplementary file 1 [file Data_Sheet_1.docx]

***Supplementary Material***

**The Detrimental Effects of Delay on the Endorsement of Misleading Details for Emotionally Salient Events**

**Datin Shah & Lauren Knott***

***Correspondence:** Dr Lauren Knott: [lauren.knott.1@city.ac.uk](mailto:lauren.knott.1@city.ac.uk)

1. **Supplementary Analysis: ANOVA**

We also performed an ANOVA for comparability and consistency purposes with previous research. The results were similar to those obtained with GEE. The false recognition responses to misleading and control minor details were analysed using a 3 (picture emotion: negative/high vs. negative/low vs. neutral) x 2 (detail type: central vs. peripheral) x 2 (misinformation: misled vs. control) x 2 (retention interval: immediate vs. delayed) repeated measures ANOVA. Analysis revealed a significant main effect of misinformation, *F*(1, 45) = 35.83, *p* < .001, η_p_^2^ = .44, and detail type, *F*(1, 45) = 4.66, *p* = .036, η_p_^2^ = .09. False recognition was significantly higher for misleading details (*M* = .48, *SD* = .16) compared to control details (*M* = .30, *SD* = .14) and for central details (*M* = .42, *SD* = .14) compared to peripheral details (*M* = .36, *SD* = .16). There was also a significant retention interval x misinformation interaction, *F*(1, 45) = 10.95, *p* = .002, η_p_^2^ = .20, and a picture emotion x retention interval x misinformation interaction (see Figure 3), *F*(2, 90) = 3.44, *p* = .036, η_p_^2^ = .07. There were no further significant main effects (*F*s < 3.14, *p*s > .08), two-way interactions (*F*s < 1.00, *p*s > .371), three-way interactions (*F*s < 2.28, *p*s > .138), and four-way interaction (*F* = .76, *p* = .470). The three-way interaction was decomposed at each level of picture emotion.

For the negative/high picture, there was a significant main effect of misinformation, *F*(1, 45) = 9.74, *p* = .003, η_p_^2^ = .18, but no effect of retention interval (*p* = .443) nor interaction (*p* = .492). This suggests a similar pattern for the misinformation effect at both immediate and delayed sessions and no differences in the false recognition of misleading and control details over time. For the negative/low picture, there were significant main effects of retention interval, *F*(1, 45) = 4.81, *p* = .033, η_p_^2^ = .10, and misinformation, *F*(1, 45) = 21.98, *p* < .001, η_p_^2^ = .33, which were both qualified by a significant interaction, *F*(1, 45) = 11.67, *p* = .001, η_p_^2^ = .21. Paired-samples t-tests revealed a significant misinformation effect at immediate testing (misleading: *M* = .61, *SD* = .39; control: *M* = .21, *SD* = .29), *t*(45) = 6.00, *p* < .001, *d* = 1.16, but not at delayed testing (misleading: *M* = .32, *SD* = .34; control: *M* = .28, *SD* = .31), *t*(45) = .43, *p* = .667, *d* = .10. There appears to be a decrease in false recognition of the misleading details over time. For the neutral picture, there was a significant main effect of misinformation, *F*(1, 45) = 6.85, *p* = .012, η_p_^2^ = .13, but not retention interval, *F*(1, 45) = .10, *p* = .752, η_p_^2^ = .002. However, there was a significant interaction, *F*(1, 45) = 11.45, *p* = .001, η_p_^2^ = .20. Similar to the negative/low picture, there was a significant misinformation effect at immediate testing (misleading: *M* = .54, *SD* = .35; control: *M* = .25, *SD* = .31), *t*(45) = 4.16, *p* < .001, *d* = .89, that disappeared when tested one-week later (misleading: *M* = .36, *SD* = .29; control: *M* = .40, *SD* = .36), *t*(45) = -.65, *p* = .522, *d* = .13. It appears that misinformation continued to influence memory performance over time for the high-arousing negative event, but for the low-arousing events, there was no significant negative impact of misinformation on memory over time; in fact, false recognition of the misleading details decreased over time. Although detail type did not interact with this effect, Table 1 suggests that this was more apparent in the peripheral compared to central detail type.

Based on Porter et al. (2003, 2010) and Van Damme and Smets (2014), differences in the endorsement of the major misleading details across negative and neutral pictures over time were investigated. To do so, we conducted a 3 (picture emotion: negative/high vs. negative/low vs. neutral) x 2 (misinformation: misled vs. control) x 2 (retention interval: immediate vs. delayed) mixed-factors ANOVA, with between-subjects on the last factor. There was a significant misinformation effect (misleading: *M* = .43, *SD* = .34; control: *M* = .21, *SD* = .23), *F*(1, 44) = 16.22, *p* < .001, η_p_^2^ = .27. Furthermore, there was also a significant misinformation x retention interval interaction, *F*(1, 44) = 9.19, *p* = .004, η_p_^2^ = .17. At immediate testing, accuracy was lower for misleading major details (*M* = .53, *SD* = .35) compared to control major details (*M* = .14, *SD* = .20), *t*(21) = 5.05, *p* < .001, *d* =1.38. However, this misinformation effect was no longer significant at delayed testing (misleading: *M* = .33, *SD* = .33; control: *M* = .28, *SD* = .25), *t*(23) = .70, *p* = .491, *d* = .19. There were no further significant main effects (*F*s < .380, *p*s > .668), two-way interactions (*F*s < 1.09, *p*s > .340), and three-way interaction (*F* = .15, *p* = .866).
